# Modeling the current spatial distribution of snail intermediate hosts of schistosomiasis using Maxent software in the Volta Basin (Burkina Faso)

**DOI:** 10.1016/j.parepi.2026.e00521

**Published:** 2026-06-15

**Authors:** Noellie Winkom Kpoda, Salam Sankara, Idrissa Ouédraogo, Noellie Débora Balima, Awa Gneme, Adama Oueda, Herman Sorgho

**Affiliations:** a Animal Biology and Ecology Laboratory, Joseph Ki-Zerbo University 09, B.P., 293, Ouagadougou 09, Burkina Faso; b Thomas Sankara University, 12, B.P. 417, Ouagadougou 12, Burkina Faso; c National Centre for Scientific and Technological Research, 03, B.P. 7047, Ouagadougou 03, Burkina Faso

**Keywords:** Snails intermediate hosts, Schistosomiasis, Maxent software, Volta Basin

## Abstract

**Background:**

The socioeconomic consequences of schistosomiasis have forced the integration of control strategies. Currently, all control efforts have tended to involve the control of snail intermediate hosts. However, the integration of snail control into the general schistosomiasis control strategy has not yet been effective in Burkina Faso. While snail occurrence data have been collected intermittently since the last systematic assessment in 1980, there has been no recent synthesis of the distribution of intermediate host snails in Burkina Faso. The aim of this study was to integrate historical and recent datasets to assess temporal shifts in species distribution, identify key climatic drivers, and produce spatially explicit risk maps to support targeted control strategies in Burkina Faso.

**Methods:**

Snail occurrence data were compiled for three time periods: 1980, 2018, and 2021. Data from 1980 and 2018 were obtained through a comprehensive literature review, while field sampling was conducted in 2021 in two basins: the Mouhoun basin and the Nakanbé basin. The Maximum Entropy (Maxent) modeling approach was used to predict changes in species distributions over time by integrating occurrence records from each period with relevant environmental variables. Model performance was evaluated using the Area Under the Curve (AUC) of the Receiver Operating Characteristic (ROC) curve and the Boyce index. The contribution of each environmental variable was assessed using a jackknife test.

**Results:**

Five schistosomiasis intermediate hosts were collected: *Bulinus truncatus, Bulinus forskalii, Bulinus senegalensis, Bulinus globosus,* and *Biomphalaria pfeifferi*. The number of snail intermediate host species for schistosomes did not significantly change between 1980 and 2021. Except for *B. umbilicatus*, the results show that the two basins host almost the same species of snail intermediate host of schistosomes. The rivers, reservoirs, and irrigated plains provide favorable conditions for the occurrence of all species of snail intermediate hosts. The area under the curve (AUC) of the selected variables ranged from 0.877 to 0.985. Except for *B. globosus*, the Boyce index ranged from 0.6 to 0.924. Spatial distribution modeling indicated that the western part of the Volta Basin is more suitable for the occurrence of schistosome intermediate host snails.

**Conclusions:**

Schistosomiasis intermediate host snails are common in the Nakanbé and Mouhoun basins, where suitable habitats are widespread. The Maxent model successfully identified areas with high environmental suitability for these intermediate host snails. This study integrated historical and recent occurrence data to assess temporal changes in intermediate host snail distribution, identify key climatic drivers of distribution shifts over the past 40 years, and highlight priority areas for targeted snail control in Burkina Faso.

## 1 Introduction

Schistosomiasis is a chronic and debilitating parasitic disease caused by infection with blood flukes of the genus *Schistosoma*. It affects at least 240 million people worldwide, with the majority of cases occurring in West Africa, including Burkina Faso (WHO, 2013). Transmission of schistosomiasis depends on the presence of freshwater snail species that act as intermediate hosts. The spatial and temporal distribution of the disease is thus closely tied to the ecology and distribution of these snails (Poda et al., 1994; Sangwan et al., 2016). Despite over a decade of large-scale control efforts based primarily on mass drug administration (MDA) with praziquantel (PZQ), schistosomiasis remains widespread across Africa. In Burkina Faso, high transmission persists in several regions (Sokolow et al., 2016). While PZQ is effective at clearing existing infections, it does not prevent reinfection by cercariae shed from infected snails. Consequently, the global burden of schistosomiasis has only slightly changed since the drug's introduction (Tian-Bi et al., 2018). Sustainable control of the disease will require interrupting transmission by targeting both the human and snail hosts. The World Health Organization's 2021–2030 roadmap for neglected tropical diseases emphasizes the need for integrated approaches combining MDA with snail control interventions to achieve the elimination of schistosomiasis as a public health problem by 2030 (WHO, 2020). However, environmental management strategies for snail control have rarely been implemented in West Africa due to limited resources and the absence of precise information on where such interventions would be most effective. Snail control is particularly challenging in large or inaccessible water bodies (Steinmann et al., 2006), highlighting the need for tools that can help identify priority areas especially those with high human-water contact and intense transmission for targeted control.

In Burkina Faso, six species of freshwater snails have been identified as potential intermediate hosts of schistosomes. Five species (*B. truncatus*, *B. globosus*, *B. senegalensis*, *B. forskalii*, and *B. umbilicatus*) are involved in the transmission of *Schistosoma haematobium*, while *Bi. pfeifferi* is the sole intermediate host of *Schistosoma mansoni* (Poda et al., 2004). Since the last national survey conducted in 1980, available information on intermediate host snails of schistosome has remained limited to non-systematic studies, including those carried out in 2018, which report fragmented and incomplete records of their distribution. This highlights a persistent gap in up-to-date and spatially comprehensive knowledge of their distribution, potentially limiting the implementation of effective schistosomiasis control strategies (Kloos et al., 2001). Given the environmental dependence of schistosomiasis transmission (Ajakaye et al., 2016; Manz et al., 2020; Roquis et al., 2016), ecological modeling and remote sensing tools offer promising approaches to identify high risk areas. Remote sensing allows researchers to characterize environmental conditions favorable to snail habitats, even in areas not directly surveyed during epidemiological studies (Walz et al., 2015). Among ecological modeling tools, the Maximum Entropy (Maxent) model has proven particularly effective for predicting species distribution based on presence-only data and environmental variables (Phillips and Dudík, 2008; Roeck et al., 2014; Simoonga et al., 2009). It can help identify the potential distribution of intermediate host snails and inform targeted interventions in areas where schistosomiasis risk is highest (Pedersen et al., 2014).

This study aims to address the current gap in knowledge on the spatial and seasonal distribution of snail intermediate hosts in Burkina Faso. Using Maxent modeling and environmental data, we assess habitat suitability for six key snail species in the central and western regions of the country. Our goal is to provide updated on species distribution by integrating historical and recent datasets to analyse temporal shifts over four decades, identify keys climatic drivers of these changes, and provide spatially explicit risk maps that can support targeted control strategies in Burkina Faso.

## 2 Material and methods

### 2.1 Study areas

**Fig. 1:**
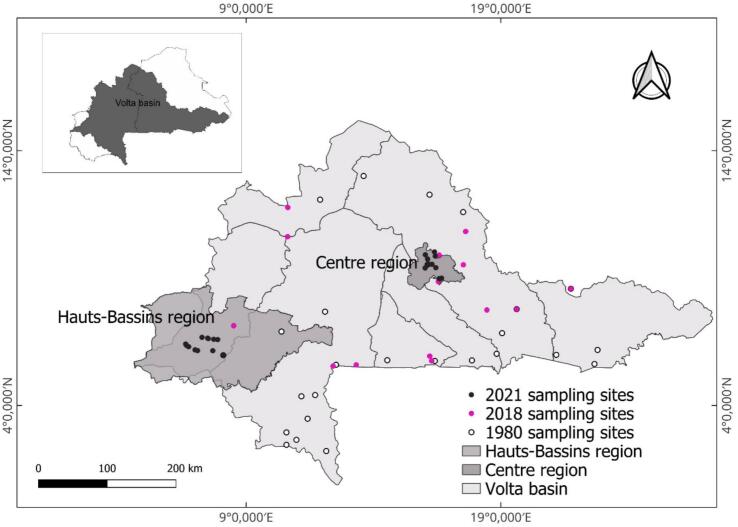
Map of the sampling areas.

This study was conducted in the Volta Basin of Burkina Faso, specifically within two administrative regions: the Hauts-Bassins and the Centre regions (Fig. 1). These regions are located within two different climatic zones of the Volta Basin: the Sudanian and the Sudano-Sahelian zones, respectively. Burkina Faso's climate is characterized by two distinct seasons: a rainy season (June to September) and a dry season (October to May) (Thiombiano et al., 2006). Based on climatic data from the Agence Nationale de la Météorologie (ANAM) (2012), the country is divided into three main climatic zones:

- The Sudanian zone, located in the southern part of the country, receives more than 900 mm of annual rainfall.
- The Sudano-Sahelian zone, covering the central region, receives between 600 and 900 mm of rainfall annually.
- The Sahelian zone, in the north, receives less than 600 mm of rainfall.

The Hauts-Bassins and Centre regions were selected because they are both located in the Volta Basin and are endemic zones for schistosomiasis. The selection of these study sites was based on previous epidemiological data, accessibility, presence of human-water contact sites, and diversity of water bodies relevant to snail habitats (reservoirs, rivers, and irrigated plains).

#### 2.1.1 Hauts-Bassins region

Located in the western part of Burkina Faso, the Hauts-Bassins region covers an area of 25,574 km^2^ (9.4% of the national territory). It includes major hydrographic networks flowing towards Côte d'Ivoire, with the Samendéni reservoir being the largest in the region. The climate is predominantly Sudanian with annual rainfall exceeding 900 mm. The region is known for agricultural activities surrounding Bobo-Dioulasso and includes two major irrigated plains Banzon and Kou Valley, which have been associated with high transmission of schistosomiasis (Poda et al., 2003; Steinmann et al., 2006; Yapi et al., 2017). According to the Ministry of Health (2018), schistosomiasis prevalence remains high in this region, with some localities reaching up to 25%. *S. mansoni* is now more dominant than *S. haematobium* (Bagayan et al., 2016; Kpoda et al., 2013; Zongo et al., 2013). Snail species previously reported in the region include *B. globosus*, *Bi. pfeifferi*, *B. truncatus*, *B. senegalensis*, and *B. forskalii* (Poda et al., 1994).

#### 2.1.2 Centre region

The Centre region is located in the Sudano-Sahelian zone and covers approximately 2869 km^2^. Rainfall ranges between 600 and 900 mm annually, and the average annual temperature is around 29.6 °C. The main hydrographic feature is the Nazinon River, supplemented by numerous temporary water bodies and artificial reservoirs, especially in peri-urban areas around Ouagadougou and the Koubri locality. These water bodies are frequently used for irrigation, horticulture, fishing, and domestic purposes, and are often polluted by household and industrial waste. The region was part of the national schistosomiasis elimination strategy from 2015 to 2020, with encouraging results including a significant drop in prevalence in some areas (Bagayan et al., 2014). Malacological studies reported the presence of *B. globosus*, *Bi. pfeifferi*, *B. truncatus*, *B. senegalensis*, and *B. forskalii* (Poda et al., 1994).

### 2.2 Sampling method

This study used snail occurrence data from three time periods: 1980, 2018, and 2021. Historical data (1980 and 2018) were obtained through an extensive literature review of previous malacological surveys conducted in the Volta Basin. From November 2020 to September 2021, a field survey was carried out in the same basin, targeting the Hauts-Bassins and Centre regions. At each site, several sampling points were identified based on accessibility. At each station, snails were collected by two experienced collectors using scoops and forceps to inspect all microhabitats (floating objects, mud, aquatic plants, rocks) within a water body for 15 min. For the deep points, snails were collected using an Ekman grab. The collected snails were rinsed and preserved in ethanol, then transported to the laboratory. In the laboratory, species identification was conducted through morphological examination of the shell, using the identification key provided by Danish Bilharziasis Laboratory (1981).

Species richness was defined as the total number of schistosomiasis intermediate host species recorded for each basin and survey year. Analyses were carried out across the two basins that constitute the Volta Basin. Spatial filtering was performed on all occurrence data in order to retain only one presence point per 1 × 1 km grid, corresponding to the resolution of the environmental variable layers (Luis et al., 2018). We also assessed spatial autocorrelation among occurrence records using average nearest neighbour analyses implemented in the spThin (version 0.2.0) package in R (version 4.4.2) (R Core Team, 2024) to remove spatially correlated points (Bosso et al., 2016). Although these approaches help reduce spatial bias in the presence dataset, they do not fully account for sampling effort in presence-only models such as Maxent (Fourcade et al., 2014). Then, only species with ten or more unique occurrence points per period were selected (Table 1), as recommended for modeling reliability (Wisz et al., 2008). All occurrence data were georeferenced and compiled into a .csv file.

**Table 1:** Number of occurrence records used for Maxent modeling by survey year.

Snail Species	1980	2018	2021
*B. truncatus*	16	3⁎	15
*B. globosus*	17	4⁎	13
*B. senegalensis*	12	7⁎	18
*B. forskalii*	18	4⁎	19
*Bi. pfeifferi*	15	6⁎	15
*B. umbilicatus*	5⁎	2⁎	0⁎

⁎ Maxent distribution not applied to these sites for low number of occurrence points.

#### 2.2.1 Environmental layers

**Fig. 2:**
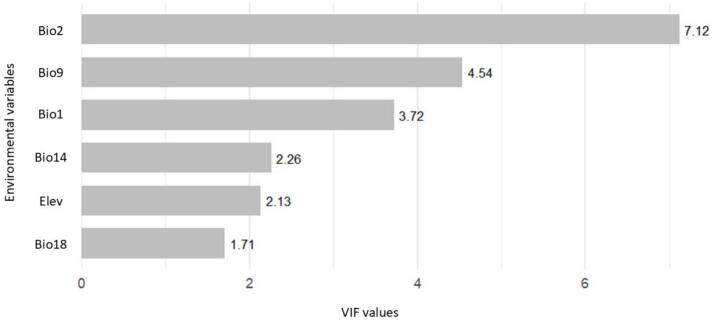
Environmental variables selected according to VIF values.

Bioclimatic variables (temperature and precipitation) were selected based on their known influence on the bio-ecology of intermediate hosts snails (Appleton, 1977; Stensgaard et al., 2013). However, we were unable to include other important variables that can influence the distribution such as land cover, water quality, and distance to water (Nkolokosa et al., 2025; Nwoko et al., 2022), which represents a limitation of this study. Bioclimatic variables were downloaded from raw data from WorldClim version 2.1 (Hijmans et al., 2005) at a resolution of 30 arc *sec* (approximately 1km^2^). These data, in “.tif” format, were processed and transformed into “.asc” format for use in Maxent version 3.4.0. Variance inflation factor (VIF) was used to assess for multicollinearity among variables using usdm version 2.1–7 package. Variables with VIF value >10, were not included as it indicated that a predictor's variance is inflated due to its correlation with other predictors, making its coefficient estimates unreliable and leading to difficulties in determining the unique impact of individual variables (Gaudreau et al., 2015). This procedure was used until all selected variables achieved VIF of less than 10 (Fig. 2).

Because of these two approaches, six environmental variables were selected (Table 2).

**Table 2:** List of environmental variables using for Maxent modeling.

Environment Variables	Variables	Codes	Description	Sources	Resolution
	wc2.1_30s_bio_1	Bio1	Annual mean temperature	WorldClim 2.1	1 km
	wc2.1_30s_bio_2	Bio2	Mean diurnal range: mean of monthly (max. Temp - min. temp)	WorldClim 2.1	1 km
Bioclimatic	wc2.1_30s_bio_9	Bio9	Mean temperature of driest quarter	WorldClim 2.1	1 km
	wc2.1_30s_bio_14	Bio14	Precipitation of driest month	WorldClim 2.1	1 km
	wc2.1_30s_bio_18	Bio18	Precipitation of warmest quarter	WorldClim 2.1	1 km
Topographic	wc2.1_30s_elev	elev	Elevation	SRTM (Shuttle Radar Topography Mission)	250 m

#### 2.2.2 Model implementation

During this study, the maximum entropy theory software (Maxent) was used to predict the suitable habitat of snail intermediate hosts according to bioclimatic variables. Maxent is a useful tool for identifying areas with high chances of finding a species. Its advantage is that only environmental variables and presence points are required to provide more accurate predictions even with small sample sizes (Pearson et al., 2006; Phillips and Dudík, 2008; Saupe et al., 2015). The entropy model was found to be the best in terms of predictive performance and model stability compared to other similar niche models (Phillips et al., 2006) and provides a distribution closer to the realized distribution of the species (Elith et al., 2006). In this study, Maxent was selected because it is particularly suitable for presence only data and small sample size, which characterize our dataset (Phillips et al., 2006; Wisz et al., 2008). Maxent has been widely used for ecological niche modeling of freshwater snails and schistosomiasis transmission (Manyangadze et al., 2016; Stensgaard et al., 2013) and has demonstrated robust performance compared to alternative algorithms in similar contexts. All models were built randomly using 75% of the species distribution points for training, and the remaining 25% were used for evaluation. We used a boostrap with 10 replications, and the mean results were used for modeling. Model performance was assessed using the area under the receiver operating characteristic curve (AUC). AUC values were interpreted as follows: values between 0.5 and 0.7 indicate low accuracy, 0.7–0.9 indicate moderate accuracy, and values above 0.9 indicate high accuracy, following the method described by (Swets, 1988). The jackknife test was used to determine the importance and contribution of each environmental variable to model development. The suitability maps (by species) obtained from the logistic outputs of Maxent were imported into R software to generate species distribution maps. The suitability maps for each species were divided into four levels following the method reported in Yang et al. (2013) and Ansari and Ghoddousi (2018). The Boyce index was used to evaluate the accurate prediction of suitable habitats for the species (Barbet-Massin et al., 2012; Hirzel et al., 2006). This index ranges from 0 to 1. A value close to 1 (greater than 0.6) indicates that habitats classified as suitable correspond well to the locations where the species is actually found. In contrast, a value close to 0 (less than 0.4) indicates no relationship between the model predictions and the actual presence data (Hirzel et al., 2006).

## 3 Results

### 3.1 Species richness and occurrence of snail intermediate hosts of schistosomes in the Nakanbé and Mouhoun basins in 1980, 2018 and 2021

In the Nakanbé and Mouhoun basins, 5 intermediate host species of schistosomes were collected in 2021, while 6 species were collected in 2018 and 1980.

**Fig. 3:**
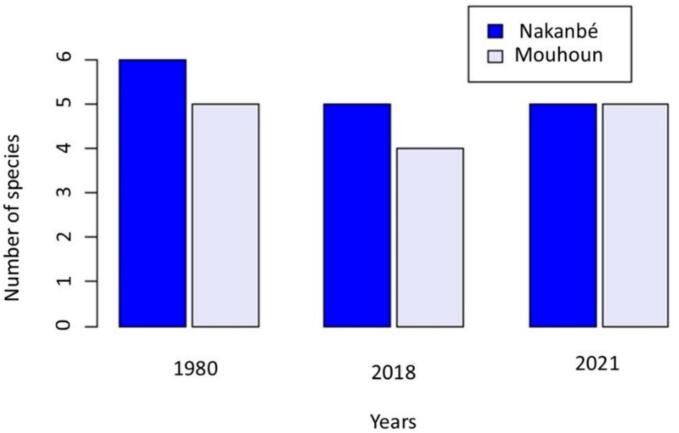
Species richness of the two basins according to the year of surveys.

Only *B. umbilicatus* was not recorded during the 2021 surveys. Fig. 3 shows the species richness of schistosome intermediate host snails in the two basins of the Volta Basin in 1980, 2018, and 2021. In the Mouhoun Basin, 5 species were collected in 1980 and 2021 while 4 species were collected in 2018. In the Nakanbé Basin, six species were recorded in 1980, compared to five species in 2018 and 2021. Table 3 shows that *B. globosus* was collected in the Nakanbé Basin in 1980 and 2021, but was not found in 2018. However, this species was found in all three campaigns in the Mouhoun Basin. *B. umbilicatus* is a species exclusively found in the Nakanbé Basin. In the Mouhoun Basin, it did not report (Table 3). *B. forskalii, B. senegalensis, and Bi. pfeifferi* were consistently present in both basins*.*

**Table 3:** Occurrence of snail species in the Nakanbé and Mouhoun basins.

Years	Species	Volta Basin		References
		Mouhoun	Nakanbé	
1980	*B. umbilicatus*	–	⁎	(Poda et al., 1994; Poda and Traoré, 2000)
	*B. forskalii*	⁎	⁎	(Poda et al., 1994)
	*B. globosus*	⁎	⁎	(Poda et al., 1994)
	*B. truncatus*	⁎	⁎	(Poda et al., 1994)
	*B. senegalensis*	⁎	⁎	(Poda et al., 1994)
	*Bi. pfeifferi*	⁎	⁎	(Poda et al., 1994)
2018	*Bi. pfeifferi*	⁎	⁎	(Ouédraogo, 2018)
	*B. umbilicatus*	–	⁎	(Ouédraogo, 2018)
	*B. forskalii*	⁎	⁎	(Ouédraogo, 2018)
	*B. globosus*	⁎	–	(Ouédraogo, 2018)
	*B. senegalensis*	⁎	⁎	(Ouédraogo, 2018)
	*B. truncatus*	–	⁎	(Ouédraogo, 2018)
2022	*Bi. pfeifferi*	⁎	⁎	
	*B. forskalii*	⁎	⁎	
	*B. globosus*	⁎	⁎	This study
	*B. senegalensis*	⁎	⁎	
	*B. truncatus*	⁎	⁎	

⁎ = species found, − = no species found.

### 3.2 Species richness and occurrence of the intermediate snail hosts of schistosomes by habitat in 1980, 2018 and 2021

Reservoirs and rivers are two of the main preferred habitats for snail intermediate hosts of schistosomes in Burkina Faso. Table 4 shows the occurrence of intermediates host snail species of schistosomes by habitat type during the three collection periods. The 1980 surveys showed that all species were present in both reservoirs and rivers. In 2018, all six species were recorded in reservoirs, while four species were recorded in streams. *B. umbilicatus* and *B. truncatus* were not found in the rivers. The findings of the 2021 survey showed that, with the exception of *B. umbilicatus,* all the 5 intermediate host species were found in both reservoirs and rivers.

**Table 4:** Occurrence of snail intermediate host species of schistosomes according to habitat type.

Years	Species	Habitats		References
		Reservoirs	Rivers	
1980	*Bi. pfeifferi*	⁎	⁎	(Poda et al., 2004; Poda and Traoré, 2000)
	*B. senegalensis*	⁎	⁎	(Poda et al., 2004; Poda and Traoré, 2000)
	*B. truncatus*	⁎	⁎	(Poda et al., 2004; Poda and Traoré, 2000)
	*B. umbilicatus*	⁎	⁎	(Poda et al., 2004; Poda and Traoré, 2000)
	*B. forskalii*	⁎	⁎	(Poda et al., 2004; Poda and Traoré, 2000)
	*B. globosus*	⁎	⁎	(Poda et al., 2004; Poda and Traoré, 2000)
2018	*Bi. pfeifferi*	⁎	⁎	(Ouédraogo, 2018)
	*B. umbilicatus*	⁎	**–**	(Ouédraogo, 2018)
	*B. forskalii*	⁎	⁎	(Ouédraogo, 2018)
	*B. globosus*	⁎	⁎	(Ouédraogo, 2018)
	*B. senegalensis*	⁎	⁎	(Ouédraogo, 2018)
	*B. truncatus*	⁎	**–**	(Ouédraogo, 2018)
2022	*B. forskalii*	⁎	⁎	
	*B. globosus*	⁎	⁎	
	*Bi. pfeifferi*	⁎	⁎	This study
	*B. senegalensis*	⁎	⁎	
	*B. truncatus*	⁎	⁎	

⁎ = species found, − = no species found.

### 3.3 Modeling of the spatial distribution dynamics of snail intermediate hosts of schistosomes

**Fig. 4:**
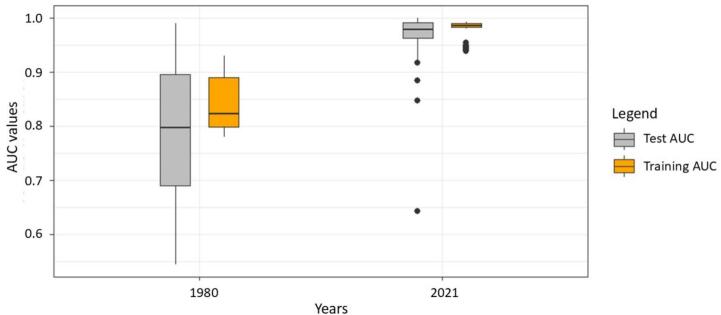
AUC values according to training and test.

The Maxent model showed good performance for both years. In 1980, the Maxent model performed well, with median values of 0.823 and 0.797 for training and testing, respectively. In 2021, the Maxent model also demonstrated good performance, with median values of 0.986 and 0.979 for training and testing, respectively (Fig. 4).

For 1980, the mean AUC values ranged from 0.763 ± 0.130 for *B. globosus* to 0.815 ± 0.122 for *B. forskalii*. The Boyce index ranged from 0.73 to 0.924, indicating a good ability to detect species presence. For 2021, the AUC values ranged from 0.903 ± 0.090 for *B. globosus* to 0.966 ± 0.043 for *B. forskalii*. The Boyce index ranged from 0.187 to 0.903, indicating a low detection ability for *B. globosus* (0.187) and a good detection ability for the other species (Table 5). The distribution of intermediate snail hosts is primarily related to variations in precipitation and temperature (Table 5). For 1980, the results showed that the distribution of intermediate host species was influenced by two main bioclimatic variables. The distribution of *B. globosus* and *Bi. pfeifferi* was strongly influenced by the average temperature of the driest quarter (Bio9). The adequacy between the occurrence of these species and the increase in temperature reached a maximum at 29 °C. The distribution of *B. forskalii*, *B. truncatus*, and *B. senegalensis* was strongly influenced by precipitation in the warmest quarter (Bio18), with suitability peaking at around 250 mm (additionale file Figs. S1-S5). In 2021, the mean diurnal temperature range (Bio2) influenced the distribution of all intermediate host snail species. The probability of species occurrence decreased as the average daily temperature range increased, up to 17 °C. The influence of other variables varied across species. The distribution of *B. truncatus* and *B. senegalensis* was influenced by the mean temperature of the driest quarter (Bio9) in addition to Bio2. The cumulative contribution of these two variables exceeded 60%, making them the main factors contributing to the Maxent model. The occurrence probability of these two species also decreased as temperatures increased during the dry quarter, from 26 °C to 29 °C. The distribution of *B. globosus* and *Bi. pfeifferi* was mainly influenced by Bio2 and Bio14, with a cumulative contribution greater than 67%. The occurrence probability of these species decreased as precipitation in the driest quarter increased (up to 4 mm). The distribution of *B. forskalii* was influenced by Bio2, Bio9, and Bio18, with a cumulative contribution of 78% to the model (Table 5). Its occurrence probability decreased as the mean temperature of the driest quarter increased from 26 °C to 29 °C, while suitability increased with precipitation in the warmest quarter up to 250 mm (Figs. S6–10).

**Table 5:** Contributions of bioclimatic variables determined using the Jackknife test.

			Boyce index	Contributions of environmental variables					
Years	Species	AUC		Bio1	Bio2	Bio9	Bio14	Bio18	elev
1980	*B. forskalii*	0.815 ± 0.122	0.795	8.3	5.2	9.7	19.15	43.9	13.4
	*B. globosus*	0.763 ± 0.130	0.73	2.4	0.0	41.5	22.7	21.8	11.6
	*Bi. pfeifferi*	0.792 ± 0.116	0.72	2.5	0.0	39.2	24.3	22.8	11.3
	*B. senegalensis*	0.809 ± 0.111	0.823	13.7	0.0	0.9	18	65.5	1.7
	*B.truncatus*	0.787 ± 0.128	0.924	16.6	3.5	5.8	3.7	63.8	6.4
2021	*B. forskalii*	0.966 ± 0.043	0.667	14.1	28.4	23.1	7.6	26.5	0.3
	*B. globosus*	0.903 ± 0.090	0.187	10.5	36.7	17.9	34.1	0.8	0.0
	*Bi. pfeifferi*	0.980 ± 0.012	0.643	11.7	48.8	18.1	18.9	1.7	0.8
	*B. senegalensis*	0.986 ± 0.009	0.900	15.7	37.9	24.9	5.6	13.5	2.3
	*B. truncatus*	0.983 ± 0.010	0.903	12.12	35	26.9	12.7	11.9	1.3

**Fig. 5:**
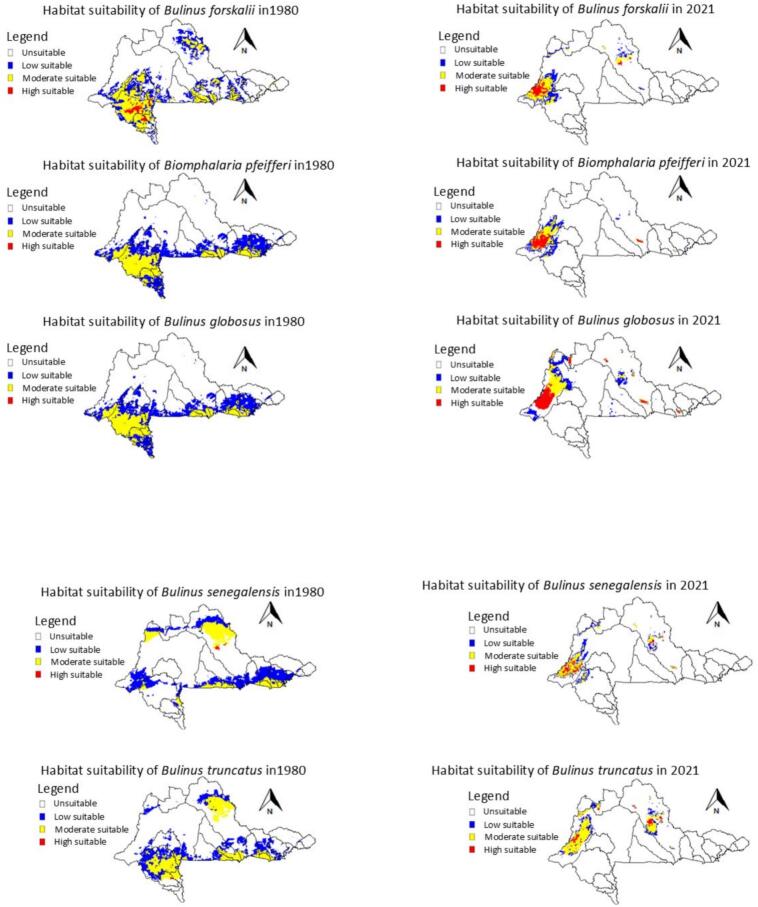
The suitable habitats of *B. truncata*, *Bi. pfeifferi, B. globosus*, *B. senegalensis* and *B. forskalii* in the Volta Basin.

The change in the potential distribution of schistosome intermediate hosts in the Volta Basin is presented in Fig. 5. In 1980, suitable habitats for the species were located mainly in the southern part of the basin. Some species, such as *B. senegalensis*, *B. forskalii*, and *B. truncatus*, also had suitable habitats in the northeastern part. For all species, unsuitable areas covered a much larger proportion of the basin compared with suitable areas. Highly suitable areas represented only a very small proportion (Table 6). In 2021, the model predicted that the southern part of the Volta Basin is a highly suitable area for intermediate snail hosts of schistosomiasis. Some species such as *B. tuncatus, B. senegalensis*, and *B. forskalii* have also shown suitable areas in the central part of the basin. Compared with 1980, unsuitable areas have increased for all species. However, highly suitable areas have increased for all species (Fig. 5 and Table 6). Furthermore, the distribution maps of 2021 also reveal an overlap of very suitable habitats for schistosome snail hosts in this part of the basin (Fig. 5). These findings show that current climatic conditions appear to favor the presence of intermediate host species of schistosomes in the western part of the Volta Basin.

**Table 6:** Change in the potential distribution of snail intermediate hosts of schistosoms between 1980 and 2021 in the Volta Basin.

		Years			
		1980		2021	
Species	Classe names	Area (km^2^)	Proportion (%)	Area (km^2^)	Proportion (%)
	Unsuitable	115,592.887	68.298	157,215.281	92.891
*Bi. pfeifferi*	Low suitable	31,441.058	18.577	4759.888	2.812
	Moderate suitable	22,186.737	13.109	4297.214	2.539
	High suitable	25.796	0.015	2974.098	1.757
	Unsuitable	114,053.413	67.388	155,702.435	91.997
*B. forskalii*	Low suitable	25,868.994	15.285	5139.347	3.036
	Moderate suitable	27,103.069	16.014	6031.411	3.564
	High suitable	2221.004	1.312	2373.286	1.402
	Unsuitable	115,783.449	68.411	150,616.344	88.992
*B. globosus*	Low suitable	31,099.878	18.375	7166.461	4.234
	Moderate suitable	22,335.692	13.197	6380.913	3.770
	High suitable	27.461	0.016	5082.762	3.003
	Unsuitable	125,160.096	73.951	158,300.402	93.532
*B. senegalensis*	Low suitable	27,332.743	16.149	4392.078	2.595
	Moderate suitable	16,538.115	9.771	5155.990	3.046
	High suitable	215.526	0.127	1398.009	0.826
	Unsuitable	122,322.470	72.274	154,041.467	91.016
*B. truncatus*	Low suitable	24,705.650	14.597	4315.521	2.549
	Moderate suitable	22,155.948	13.090	9599.662	5.672
	High suitable	62.411	0.036	1289.829	0.762

## 4 Discussion

This study provides an updated assessment of the spatial distribution of schistosomiasis intermediate host snails in Burkina Faso by integrating historical and recent occurrence data and applying species distribution modeling. The results revealed significant changes in spatial patterns, with a marked westward and northward shift in the predicted distributions of *B. globosus*, *Bi. pfeifferi*, and *B. truncatus*. The Nakanbé Basin still hosts slightly higher species richness, as initially noted by Poda (1994), likely due to the larger number of reservoirs constructed in this basin, which provide favorable habitats for host snail populations (Perez-Saez et al., 2016; Sule et al., 2025). The strong similarity in species composition between reservoirs and rivers highlights the importance of hydrological connectivity as previously noted by Perez-Saez et al. (2016). Most reservoirs in Burkina Faso are artificial and derived from river diversions, allowing passive dispersal of snails between habitat types (Boelee et al., 2010; Gerald et al., 2020; Habib et al., 2021). These artificial water bodies are often nutrient-rich, temporary, and subject to high human use, making them key transmission foci for schistosomiasis (Doumenge et al., 1987; Perez-Saez et al., 2016; Talla et al., 1992).

Our findings highlight the key role of climatic variables, particularly temperature and precipitation, in shaping habitat suitability, confirming that environmental conditions are major determinants of snail distribution patterns. In 1980, precipitation of the hottest quarter (Bio18) and mean temperature of the driest quarter (Bio9) were the most influential predictors. In 2021, the mean diurnal temperature range, Bio9, Bio14, and Bio18 remained highly important, underlining the role of thermal stress during the dry season a key constraint for aquatic snails (Brown, 1994; IPCC, 2021). For example, *Bi. pfeifferi* and *B. globosus* were strongly influenced by the diurnal temperature range and precipitation during the driest quarter (Bio14). These variables likely affect biological processes such as desiccation tolerance, reproduction cycles, and egg viability (Ahmed et al., 2021; Appleton, 1978; Sturrock, 2001). A higher diurnal range may induce water loss in snail tissues, while limited precipitation reduces the availability of aquatic habitats, especially temporary water bodies that many species rely on. Other species, such as *B. truncatus* and *B. senegalensis*, showed wider climatic tolerance, which may explain their more scattered yet resilient distributions (Manyangadze et al., 2016; Rollinson et al., 2001; van der Deure et al., 2024). *B. forskalii* was recorded in 2021 in heavily polluted watercourses, such as the Houët River in Bobo-Dioulasso. This species is known for its tolerance to eutrophic and polluted conditions, which may explain its expansion in urban and peri-urban zones (N'Goran et al., 1997; Perez-Saez et al., 2016; Pointier et al., 2005). These findings are similar to those of previous studies which highlight the importance of temperature and rainfall in predicting the distribution of schistosomiasis-transmitting snails (Ayob et al., 2025; Ayob et al., 2023; Manyangadze et al., 2016; Pedersen et al., 2014; Zhou et al., 2008). Ohter studies also demonstrated that variables such as hydrological regimes, seasonal variability, land use, and water quality strongly influence snail population dynamics and transmission patterns (Manyangadze et al., 2021; Nwoko et al., 2023; Nwoko et al., 2022; Perez-Saez et al., 2016). The predictive maps generated through Maxent offer valuable tools for targeted snail control and disease management. Zones predicted to be suitable for multiple intermediate host species particularly in western regions should be prioritized for interventions such as molluscicide application, vegetation clearance, or environmental modification (Malone et al., 1997; Simoonga et al., 2008; Steinmann et al., 2006). Integrating ecological modeling into disease surveillance systems can support early detection of high-risk zones and guide effective resource allocation (Ahmed et al., 2021; Bakuza et al., 2017). This approach aligns with the WHO's roadmap for eliminating schistosomiasis as a public health problem by 2030, which emphasizes the need for spatial risk mapping, environmental interventions, and integrated surveillance (Rollinson et al., 2013; WHO, 2022; WHO, 2020). The long-term shift in suitable snail habitats observed here suggests that climate change may be driving ecological filtering that favors generalist species like *B. truncatus* and *B. senegalensis*, while narrowing the niche of more specialized taxa such as *B. umbilicatus* (Kloos et al., 2004; Zhou et al., 2008). These dynamics could alter transmission patterns and require adaptive responses in disease control strategies. Despite the robustness of Maxent, which uses presence-only data effectively, certain limitations must be acknowledged. The absence of important predictors such as land use, water quality, local human-water contact behaviors, or hydrological connectivity may have limited the ecological realism of our models. These variables are known to strongly influence snail habitats and schistosomiasis transmission (Nkolokosa et al., 2025; Nwoko et al., 2022). Thus, while these maps indicate environmentally suitable habitats, they should not be interpreted as direct representations of current transmission risk without field validation (Frandsen and Christensen, 1984; Phillips et al., 2006; Simoonga et al., 2008). Another limitation of this study is the use of a single modeling algorithm. Although Maxent is widely recognized for its robustness with presence-only data, relying on a single model may introduce biases related to sampling effort and model structure. Ensemble modeling approaches that combine multiple algorithms often improve predictive performance and reduce uncertainty, and should therefore be considered in future studies. Future research should explore flexible ensemble and machine learning approaches that can accommodate small sample sizes rather than excluding low-occurrence data. Overall, this study contributes to filling a critical knowledge gap by providing an updated and spatially explicit synthesis of the distribution of intermediate host snails in Burkina Faso. By identifying key environmental drivers, it offers valuable insights for targeted surveillance and control strategies. Moreover, the comparison with previous studies underscores the complementarity between mechanistic ecohydrological models and correlative approaches. Integrating these frameworks in future research could significantly improve the prediction of schistosomiasis transmission risk by linking habitat suitability with hydrological dynamics, land use, local human-water contact behaviors, water quality, and seasonal variability.

## 5 Conclusions

This study integrates historical (1980) and recent (2021) occurrence data to assess temporal changes in snail distribution and provides the first updated spatially explicit habitat suitability maps for schistosomiasis-transmitting snails in the Volta Basin. Reservoirs and streams support the same species of schistosome snail intermediate host. The prediction maps show that the areas with high suitability for intermediate snail hosts of schistosomes are more concentrated in the western part of the Volta Basin. This distribution is strongly influenced by temperature and rainfall. The Volta Basin may be becoming less and less suitable for snail intermediate hosts. Given the robustness of the models obtained, the prediction maps produced here may be useful in developing managements programs for schistosomiasis in the Volta Basin.

## Availability of data and materials

The datasets used and/or analysed during the current study are available from the corresponding author on reasonable request.

## CRediT authorship contribution statement

**Noellie Winkom Kpoda:** Writing – review & editing, Funding acquisition, Formal analysis, Conceptualization. **Salam Sankara:** Writing – original draft, Software, Methodology, Formal analysis, Data curation, Conceptualization. **Idrissa Ouédraogo:** Writing – review & editing, Conceptualization. **Noellie Débora Balima:** Investigation, Data curation. **Awa Gneme:** Writing – review & editing, Validation. **Adama Oueda:** Writing – review & editing, Validation. **Herman Sorgho:** Writing – review & editing, Validation, Supervision.

## Consent for publication

Not applicable.

## Ethical approval and consent to participate

Not applicable.

## Funding

This work was partially supported by 10.13039/501100002222the World Academy of Sciences.

## Declaration of competing interest

The authors declare that they have no known competing financial interests or personal relationships that could have appeared to influence the work reported in this paper.
